# Topoisomerase II alpha inhibition can overcome taxane-resistant prostate cancer through DNA repair pathways

**DOI:** 10.1038/s41598-021-01697-2

**Published:** 2021-11-15

**Authors:** Hiroshi Hongo, Takeo Kosaka, Yoko Suzuki, Shuji Mikami, Junichi Fukada, Mototsugu Oya

**Affiliations:** 1 Department of Urology, Keio University School of Medicine, 35 Shinanomachi, Shinjuku-ku, Tokyo, 160-8582 Japan; 2 Department of Diagnostic Pathology, Keio University Hospital, Tokyo, Japan; 3 Department of Radiology, Keio University School of Medicine, Tokyo, Japan

**Keywords:** Cancer therapy, Urological cancer

## Abstract

Cabazitaxel (CBZ) is approved for the treatment of docetaxel-resistant castration-resistant prostate cancer (CRPC). However, its efficacy against CRPC is limited, and there are no effective treatments for CBZ-resistant CRPC. This study explored the optimal treatment for CRPC in the post-cabazitaxel setting. PC3 (CBZ-sensitive) and PC3CR cells (CBZ-resistant) were used in this study. We performed in silico drug screening for candidate drugs that could reprogram the gene expression signature of PC3CR cells. The in vivo effect of the drug combination was tested in xenograft mice models. We identified etoposide (VP16) as a promising treatment candidate for CBZ-resistant CRPC. The WST assay revealed that VP16 had a significant antitumor effect on PC3CR cells. PC3CR cells exhibited significantly higher topoisomerase II alpha (TOP2A) expression than PC3 cells. Higher TOP2A expression was a poor prognostic factor in The Cancer Genome Atlas prostate cancer cohort. In the Fred Hutchinson Cancer Research Center dataset, docetaxel-exposed tissues and metastatic tumors had higher TOP2A expression. In addition, VP16 significantly inhibited the growth of tumors generated from both cell lines. Based on these findings, VP16-based chemotherapy may be an optimal treatment for CPRC in the post-CBZ setting.

## Introduction

Prostate cancer is the most prevalent cancer and second leading cause of cancer-related death among American men^1^. The standard treatment for metastatic prostate cancer is androgen deprivation therapy (ADT) using luteinizing hormone-releasing hormone analogs and antiandrogens. Although most patients with metastatic prostate cancer respond to ADT, acquired resistance is inevitable, and these lesions progress to castration-resistant prostate cancer (CRPC). Cabazitaxel (CBZ) is a derivative of docetaxel (DOC), which has cytotoxic effects against DOC-resistant prostate cancer. However, the duration of the response to CBZ is limited to only a few months^2,3^, and there are no life-prolonging options for CBZ-resistant prostate cancer. Therefore, it is an urgent task to establish a novel treatment strategy for CBZ-resistant prostate cancer.

We previously reported that reactive oxygen species (ROS) induction production was associated with the cytotoxicity of CBZ, and SESN3 contributed to CBZ resistance by regulating ROS production^4^. Moreover, we established a CBZ-resistant prostate cancer cell line and reported that signals associated with cell proliferation such as the PI3K/AKT/mTOR or MEK/ERK axis^3^ were also associated with CBZ resistance. However, these findings have not been applied clinically because compounds targeting SESN3, PI3K/AKT/mTOR, or MEK/ERK have not been confirmed to be effective against CBZ-resistant prostate cancer.

The Connectivity Map (CMAP) is an algorithm for analyzing gene expression data that was developed by the Broad Institute^5^. CMAP-based analysis makes it possible to compare gene expression patterns identified using the compound database with those of some biological states such as malignant tumors^6,7^. We previously identified ribavirin, a hepatitis C drug, as a candidate drug for overcoming DOC resistance in prostate cancer via in silico screening using CMAP-based analysis^8^. The aim of this study was to explore therapeutic options for prostate cancer in the post-cabazitaxel setting via in silico drug screening using gene expression data from our own CBZ-resistant cell line.

## Materials and methods

### Reagents

We used a rabbit polyclonal antibody against TOP2A (Abcam, Cambridge, UK) and mouse monoclonal antibodies against β-actin (Sigma-Aldrich, St. Louis, MO, USA) and Ki67 (Agilent, Santa Clara, CA, USA). WST reagents (Takara Bio, Kyoto, Japan) were also used.

### Cell lines and culture

DU145 and PC3 CRPC cells were obtained from the American Type Culture Collection C (Manassas, VA, USA) in January, 2016. DU145CR and PC3R were established as previously reported^3^. DU145, PC3, DU145CR, and PC3CR cells were routinely maintained in RPMI-1640 (Invitrogen, Carlsbad, CA, USA) supplemented with 10% fetal bovine serum (Dainippon Pharmaceutical, Tokyo, Japan) at 37 °C in a humidified 5% CO_2_ atmosphere.

### WST cell viability assay

WST cell viability assay was performed as previously reported^3^. PC3 and PC3CR cells were seeded on 96-well plates, allowed to attach for 24 h, and then treated with different concentrations of CBZ (FUJIFILM Wako Pure Chemical Corporation, Tokyo, Japan) and VP16 (Sigma-Aldrich). At the end of the incubation period, WST reagents were added to each well, and the cells were incubated for 1 h. Cell viability was estimated via colorimetry by measuring the color intensity in a plate reader at 570 nm.

### Murine prostate cancer xenograft model

We evaluated in vivo efficacy of etoposide (VP16) for CBZ-resistant prostate cancer xenograft mice model as previously reported^3^. 5- to 7-week-old male athymic nude BALB/c mice were castrated via scrotal incision under anesthesia and used to create a xenograft model using PC3CR cells. The cells (2 × 10^6^ cells) were suspended in 100 μl of Matrigel (Becton Dickinson Labware, Lincoln Park, NJ, USA) and subcutaneously inoculated into the mice. The mice were monitored, and tumors were measured every 4 days. To investigate the sensitivity to CBZ and VP16 in vivo for each tumor type, the mice were assigned to control, CBZ administration, and VP16 administration groups. CBZ (10 mg/kg) was administered intraperitoneally on day 1. VP16 (20 mg/kg) was administered intraperitoneally on days 1–3. On day 13, the mice were anaesthetized with sevoflurane (WAKO, Tokyo, Japan) and killed by cervical dislocation. The subcutaneous tumors were harvested. Animal care was performed in accordance with Keio University guidelines for animal experiments. The study was conducted according to the Animal Research Reporting In Vivo Experiments (ARRIVE) requirements.

### Immunohistochemistry

Immunohistochemical staining of the mice xenograft tumors was performed as previously reported^3^. After antigen retrieval with citric acid (pH 6.0), endogenous peroxidase activity was blocked with 1% hydrogen peroxide. Primary antibody (monoclonal anti-Ki67 antibody, 1:200 dilution) was applied, followed by secondary antibodies conjugated to a peroxidase-labeled dextran polymer. The immunoreaction was visualized using diaminobenzidine, and counterstaining was performed using 10% hematoxylin. The percent of cancer cells with positive Ki67 nuclear staining (Ki67 index) was calculated for each section based on more than 1000 cancer cell nuclei. Apoptosis was measured by the TUNEL assay using an in situ apoptosis detection kit (Takara Bio, Kyoto, Japan). For the TUNEL assay, we used control slides from the apoptosis detection kit as positive controls, and control slides without terminal deoxynucleotidyl transferase served as negative controls. The average number of stained cells was counted, and the apoptosis index was calculated as the average number in five areas in a × 400 field.

### Microarray gene expression analysis

Microarray and CMAP analysis were performed as previously described^8^. Total RNA was isolated from cell lines using an RNeasy Mini kit (Qiagen, Valencia, CA, USA). Gene expression profiles were determined using the GeneChip Human Gene 1.0 ST array (Affymetrix, Santa Clara, CA, USA) according to the manufacturer’s instructions. After generating single-stranded cDNA, fragmentation and sense-strand cDNA labeling were performed using an Affymetrix GeneChip WT Terminal Labeling Kit according to the manufacturer’s protocol. After hybridization, a GeneChip Fluidics Station 450 (Affymetrix) was used to wash the arrays, and scanning was performed using a GeneChip Scanner 3000 7G (Affymetrix). The raw intensity data from scanned images of the microarrays were preprocessed using Affymetrix Expression Console software. Expression intensities were stored as cell intensity (CEL) files, and the CEL files were normalized using the robust multichip average method. These datasets were filtered, and genes with an absolute fold change ≥ 2 ≤ 0.5 were identified as being differentially expressed. GO analysis to identify biological processes likely related with CBZ resistance was performed using DAVID tools. The analysis was performed using the FAC module set to high stringency. The FAC enrichment score (− log10 P-values/n) for each cluster was graphed. The enrichment score provides an indication of the biological significance of the clusters. To identify compounds that could reprogram the CBZ resistance-related genetic network, the CBZ resistance signature was estimated by calculating the twofold gene differences between PC3 and PC3CR cells, after which the probe list of the CBZ resistance signature was entered into the CMAP (http://www.broadinstitute.org/cmap/). According to the CMAP system^5–7,9^, the top 500 upregulated and downregulated probes compatible with the HG-U133A platform were used. The threshold of significance for the candidate compounds was set at P < 0.05. This microarray data set has been approved by the Gene Expression Omnibus (GEO) (http://www.ncbi.nlm.gov/geo/); its accession number is GSE 182619.

### Real-time quantitative PCR

Real-time quantitative PCR was performed as previously reported^3^. Total RNA was isolated using an RNeasy Mini kit (Qiagen, Hilden, Germany), with quantity and quality evaluated via spectrophotometry. Reverse transcription was conducted using a PrimeScript RT reagent Kit with gDNA Eraser (Takara Bio). The reaction mixture (1 μl) was then used as a template in a TaqMan Fast real-time quantitative PCR assay using TaqMan Universal PCR Master Mix and the CFX96 Touch Real-Time PCR Detection System (Bio-Rad Laboratories, Hercules, CA, USA). The primers and TaqMan probe sets (TaqMan Gene Expression Assays) for TOP2A (Hs01032137_m1) and human GAPDH (Hs99999903_m1) as an endogenous control were purchased from Applied Biosystems (sequences not disclosed). The cycling conditions were 50 °C for 10 min and 95 °C for 10 min followed by 40 cycles of 95 °C for 15 s and 60 °C for 1 min.

### Single-cell RNA-seq

10× genomics (Pleasanton, CA, USA) single-cell RNA-seq was performed according to the manufacture’s protocol^10^. Illumina cDNA libraries of PC3 and PC3CR cells were generated using the Chromium Single Cell 3′ Chip Kit V2 (10× Genomics). Illumina libraries were sequenced using HiSeq4000 (Illumina, San Diego, CA, USA) according to the manufacture method. A gene-cell expression matrix was generated by Cell Ranger 2.2.0 (10× Genomics). Cell clustering and t-distributed stochastic neighbor embedding plotting were performed using Seurat R package v3^11^.

### Cell extracts and western blotting

Western blotting was performed as previously described^3^. Whole-cell extracts were obtained using RIPA buffer composed of 50 mM Tris–HCl (pH 7.5), 150 mM NaCl, 1% NP-40, 0.5% deoxycholate, 0.1% sodium dodecyl sulfate, and protease inhibitors. For Western blots, 50 mg of total protein were separated by sodium dodecyl sulfate–polyacrylamide gel electrophoresis using a 12.5% acrylamide gel and transferred to a nitrocellulose membrane. Blots were incubated with peroxidase-labeled secondary antibody (Dako^®^). Signals were detected using enhanced chemiluminescence reagents with a detection system (ECL Plus™ Western Blotting Detection System, Pierce^®^) and analyzed. Intensity was quantified using an LAS 3000 system (Fujifilm, Tokyo, Japan).

### Irradiation

The effect of radiation exposure on prostate cancer cells was analyzed as previously described^12^. X-ray irradiation was delivered using an MBR-1520R-4 system (Hitachi Power Solutions, Ibaraki, Japan) at settings of 150 kV and 20 mA. The dose rate of radiation was 1.45 Gy/min. Cells were exposed to 0 or 4 Gy of ionizing radiation and fixed 30 min later with 4% paraformaldehyde.

### Data analysis of prostate cancer cohorts

Data analysis of prostate cancer cohorts was performed as previously reported^13^. Recurrence-free survival and the mRNA expression of TOP2A in TCGA prostate cancer dataset^14^ were extracted from cBioPortal (http://www.cbioportal.org/). The prognostic significance of TOP2A expression was examined using Kaplan–Meier survival analysis, and recurrence-free survival was compared using the log-rank test. Information on chemotherapy and TOP2A mRNA expression in the FH Cancer Research Center prostate cancer dataset^15^ was also extracted from cBioPortal (http://www.cbioportal.org/). Differences in TOP2A expression between before and after chemotherapy and between localized and metastatic tumors were analyzed using a *t*-test.

### Next-generation whole exome sequencing

Genomic DNA was extracted from PC3 and PC3CR cells using DNeasy Blood & Tissue Kit (Qiagen, Valencia, CA) by following manufacturer’s protocol.

Whole exome sequencing was performed by GENEWIZ (South Plainfield, NJ, USA). Exome sequencing libraries were constructed using SureSelect Target Enrichment System (Agilent Technologies, Santa Clara, CA, USA). T the libraries with different indices were mixed and loaded on Illumina HiSeq (Illumina, San Diego, CA, USA) for 2 × 150 paired-end (PE) sequencing. The raw data of exome sequencing were analyzed by bioinformatics analysts of GENEWIZ. Removing adaptor sequences, PCR primers, sequences with N bases more than 10%, and bases of quality lower than 20 by Cutadapt. Aligning clean data with reference genome by DRAGEN or BWA. The DRAGEN Genome Pipeline or GATK haplotypecaller were used to call Germline SNV/InDel and the variants were annotated by Annovar. Somatic variation analysis was performed by DRAGEN Somatic Pipeline when necessary. Detecting CNV in matched samples by ControlFREEC.

### Statistical analysis

Experiments were conducted three or more replicates. Statistical analysis was performed using Student’s *t*-test and the Tukey–Kramer method for multiple comparisons. P < 0.05 denoted statistical significance.

### Ethics approval and consent to participate

The animal experiments were approved by the Keio University Institutional Animal Care and Use Committee.

## Results

### Identification of etoposide (VP16) as a candidate treatment for cabazitaxel-resistant prostate cancer via in silico screening

**Figure 1:**
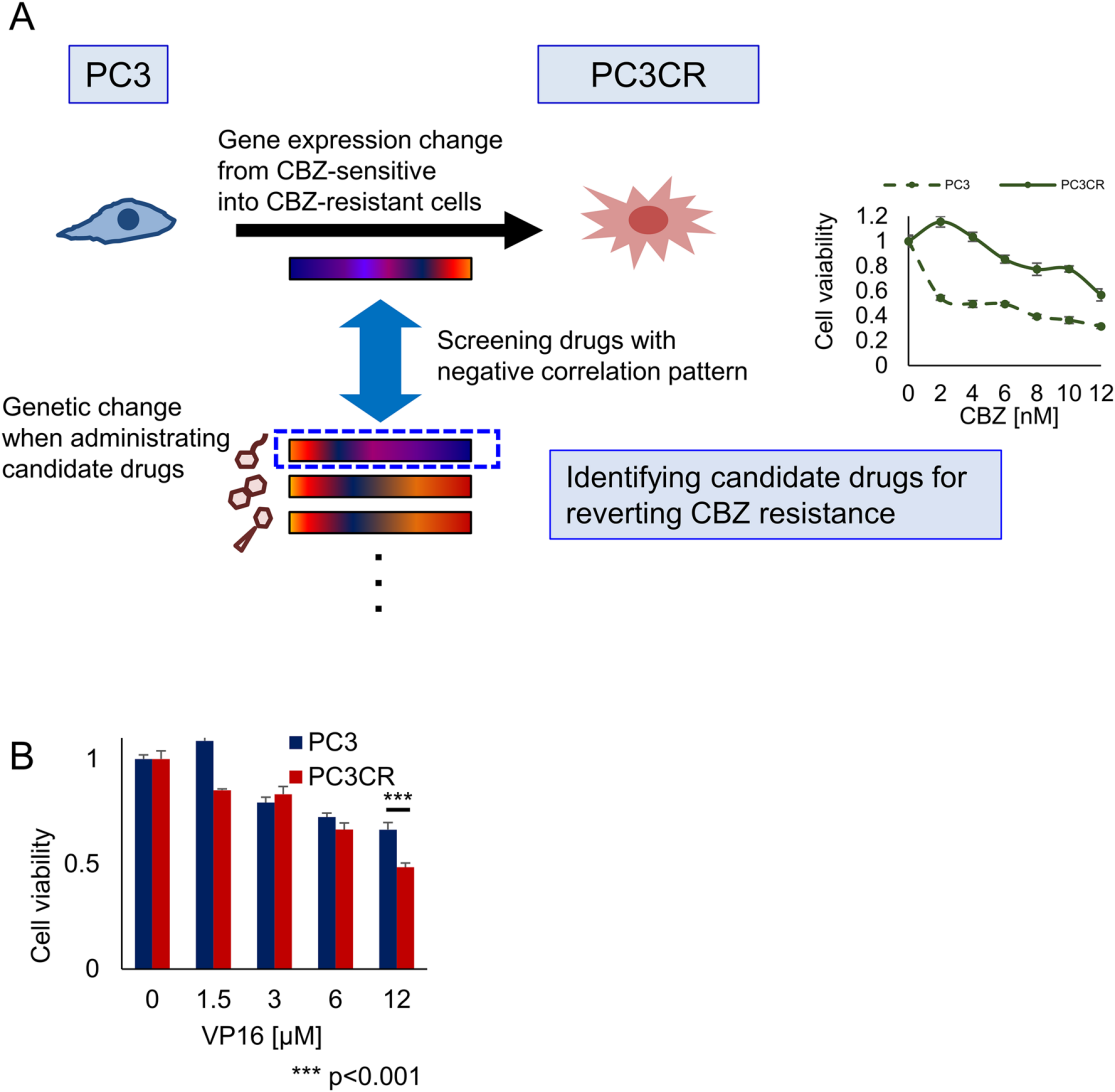
Identification of etoposide (VP16) as a candidate drug for overcoming cabazitaxel (CBZ) resistance in prostate cancer. (**A**) Schema of drug screening to overcome CBZ resistance in prostate cancer using a Connectivity Map. (**B**) On the WST assay, the relative viability of CBZ-resistant PC3CR cells treated with various concentrations of VP16 was significantly lower than that of CBZ-sensitive PC3 cells.

Using our previously established CBZ-resistant cell line PC3CR, which was generated by incubating PC3 cells with gradually increasing concentrations of CBZ, we analyzed gene expression differences between PC3 and PC3CR cells using microarray analysis. Functional annotation clustering (FAC) analysis of DAVID identified cell division (Gene Ontology [GO]: 0051301) and mitotic nuclear division (GO: 0007067) as the most enhanced clusters in PC3CR cells compared with the findings in PC3 cells (Supplementary Fig. S1). Using microarray data for PC3 and PC3CR cells, we performed CMAP-based analysis to screen for candidate drugs that reprogram the gene signature in PC3CR cells (Fig. 1A). We tested the antitumor effect of the identified candidate drugs (Table 1) in PC3 and PC3CR cells in vitro. Among the drugs, ouabain, nifedipine, benzylpenicillin, and paromomycin did not significantly inhibit PC3CR cell proliferation (Supplementary Fig. S2). Conversely, the topoisomerase II alpha (TOP2A) inhibitor VP16 exerted stronger antitumor effects on PC3CR cells than on PC3 cells (P < 0.001, Fig. 1B). Our another CBZ-resistant cell line, DU145CR derived from DU145^3^ also had higher sensitivity for VP16 than parental DU145 cells (Supplementary Fig. S3). Because VP16 has been used to treat neuroendocrine prostate cancer (NEPC), we evaluated neuroendocrine markers in prostate cancer cell lines. As previously reported^16^, NCIH660 (a NEPC model cell line) tumors expressed neuroendocrine markers such as CD56, chromogranin A and synaptophysin. Meanwhile, these markers were less strongly expressed in CBZ-resistant tumors (Supplementary Fig. S4).

**Table 1:** The list of candidate compounds for treating cabazitaxel-resistant prostate cancer.

Candidate compound	Mean	Enrichment	P-value	Specificity
Ouabain	− 0.627	− 0.948	0.0001	0.0456
Benzylpenicillin	− 0.442	− 0.782	0.00112	0.0156
Nifedipine	− 0.385	− 0.923	0.0003	0.0126
Paromomyxin	− 0.339	− 0.756	0.00078	0.0652
Etoposide	− 0.276	− 0.695	0.00539	0.065
Mycophenolic acid	− 0.267	− 0.692	0.02894	0.1284
Pivmecillinam	− 0.237	− 0.813	0.01617	0.0099

### Elevated expression of TOP2A in CBZ-resistant prostate cancer

**Figure 2:**
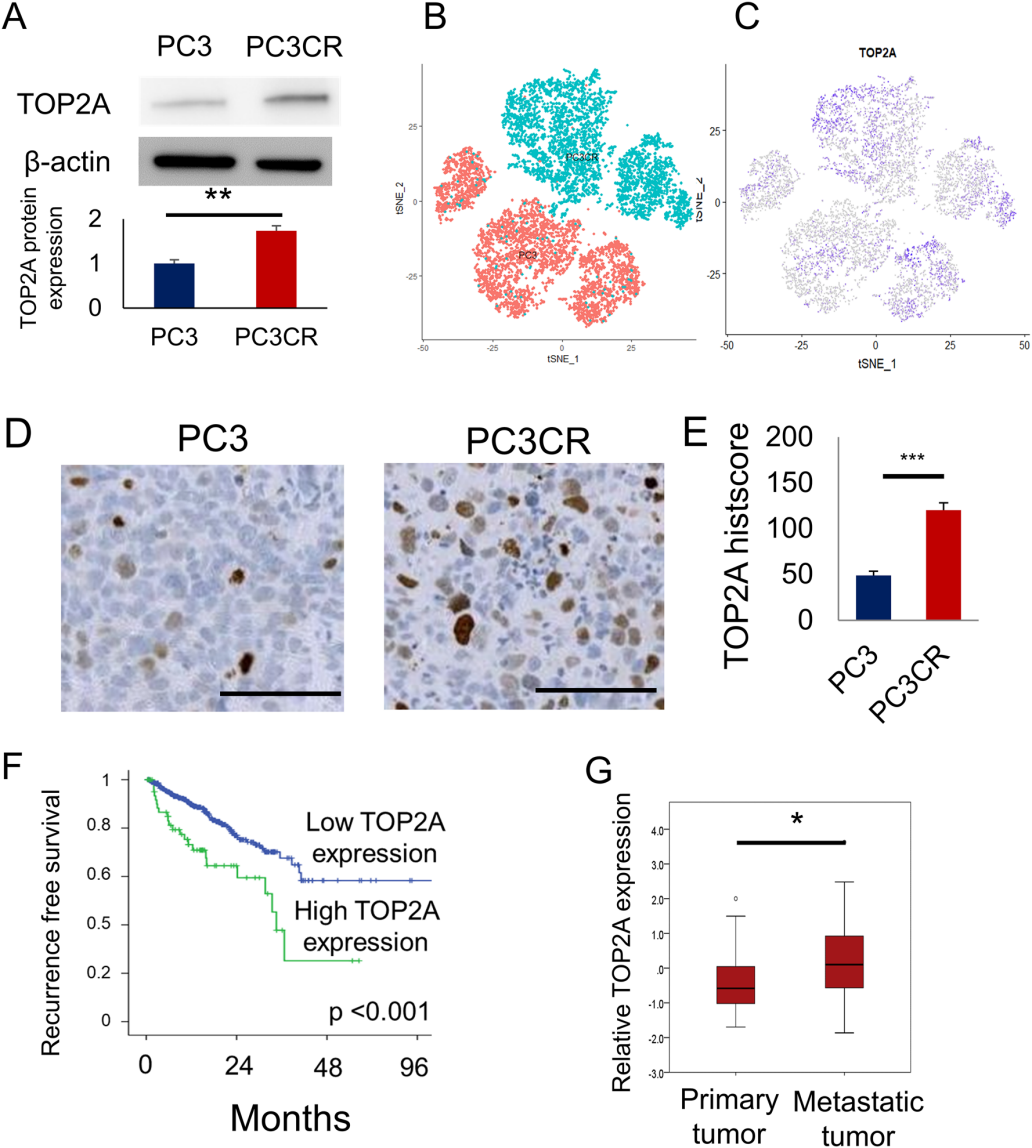
Significance of topoisomerase II alpha (TOP2A) expression. (**A**) TOP2A protein expression in PC3 and PC3CR cells. (**B**) PC3 and PC3CR cells were clustered via single-cell RNA-seq. Red dots indicate PC3 cells, and blue dots indicate PC3CR cells. (**C**) TOP2A expression was visualized using a t-distributed stochastic neighbor embedding plot. More intense purple color indicates higher TOP2A expression. (**D**) Immunohistochemical staining for TOP2A in PC3 and PC3CR xenograft tumors. Bar indicates 100 µm. (**E**) The TOP2A immunohistochemistry score was significantly higher in PC3CR tumors than in PC3 tumors. (**F**) Kaplan–Meier curves of recurrence-free survival in The Cancer Gene Atlas prostate cancer cohort according to high or low TOP2A expression. (**G**) In the Fred Hutchinson cohort, metastatic prostate cancer had significantly higher TOP2A expression than localized prostate cancer.

We analyzed TOP2A expression in CBZ-resistant cell lines. On Western blotting, TOP2A expression was higher in PC3CR cells than in PC3 cells (154.4 ± 6.8%, Fig. 2A, Supplementary Fig. S5). In single-cell RNA sequencing, the number of cells with high TOP2A expression cells was greater for PC3CR cells than for PC3 cells (Fig. 2B,C). dddNext, we evaluated TOP2A expression in CBZ-resistant xenograft tumors. On immunohistochemistry, the TOP2A histoscore in PC3CR tumors (120.2 ± 5.7) was significantly higher than that in PC3 tumors (48.8 ± 3.2, Fig. 2D,E). DU145CR also had higher TOP2A protein expression than DU145 in western blotting (Supplementary Figs. S5, S6). To investigate the clinical significance of TOP2A expression, we evaluated the correlation between TOP2A expression and prostate cancer prognosis using The Cancer Genome Atlas (TCGA) prostate cancer cohort dataset. Higher TOP2A expression was a poor prognostic factor for recurrence-free survival (p < 0.001, Fig. 2F). In the Fred Hutchinson (FH) cohort, which included patients with metastatic disease, TOP2A expression was significantly upregulated in metastatic prostate cancer tissues (Fig. 2G). These findings suggest that TOP2A is associated with the refractory nature of prostate cancer, and it could be a potential target for treating CBZ-resistant prostate cancer.

### Efficacy of VP16 for CBZ-resistant prostate cancer in vivo

**Figure 3:**
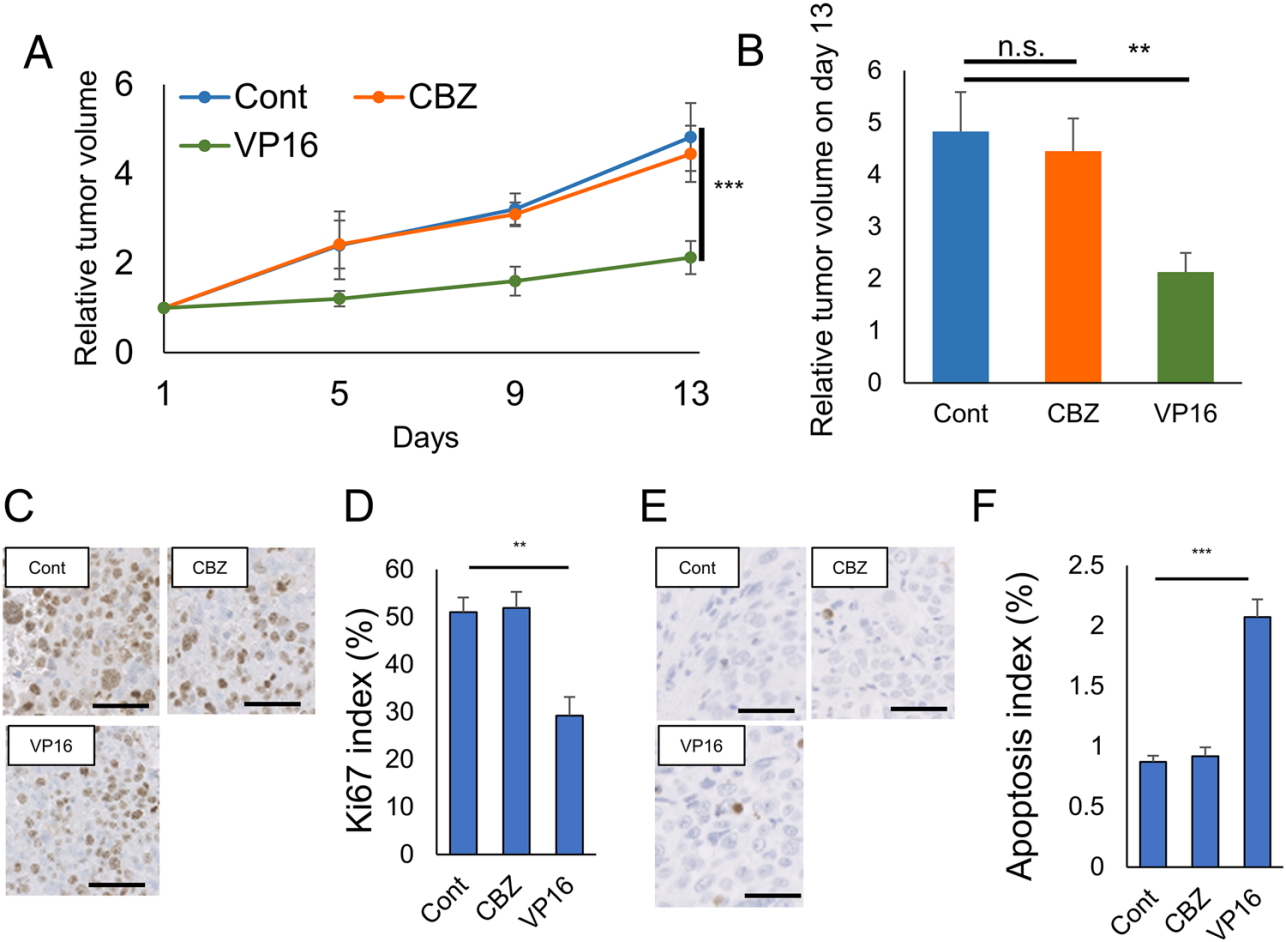
Efficacy of etoposide (VP16) in vivo. (**A**) Tumor growth over time for PC3CR xenograft tumors in castrated male nude mice during exposure to 10 mg/kg cabazitaxel (CBZ), 20 mg/kg VP16, or no treatment (Cont). (**B**) Relative tumor volume on day 13 in PC3CR xenograft tumors. (**C**) Immunohistochemical staining for Ki67 in PC3CR xenograft tumors. Bar indicates 100 µm. (**D**) Significant decrease in the Ki67 index was observed in the VP16 administration group compared with that in the control group. (**E**) TUNEL staining in PC3CR xenograft tumors. Bar indicates 100 µm. (**F**) Significant increase in the apoptosis index was noted for VP16 administration group compared with the control finding.

We determined whether VP16 exerted antitumor efficacy in PC3CR xenograft tumors. We assigned five castrated nude mice each to the control, CBZ administration (10 mg/kg on day 1), and VP16 administration groups (20 mg/kg on days 1–3). Drug administration was started when the average tumor volume was approximately 100 mm^3^. We examined time course changes in PC3CR tumor growth. Although CBZ did not significantly suppress the growth of PC3CR tumors, VP16 significantly inhibited the growth of PC3CR tumors (Fig. 3A,B). We evaluated Ki67 expression via immunohistochemistry as an index of proliferation to examine the antitumor effect of the drugs histopathologically. For PC3CR tumors, the Ki67 index was significantly lower in the VP16 administration group (35.1 ± 3.5%) than in the control group (50.3 ± 2.4%, Fig. 3C,D,F). We next evaluated apoptosis via immunohistochemistry using the TUNEL assay. In PC3CR tumors, the mean apoptosis index of the VP16 administration group (2.03 ± 0.42%) was significantly higher than that of the control group (0.86 ± 0.02%, Fig. 3E,F). These results indicated that VP16 exerted antitumor effects on CBZ-resistant tumors by inhibiting cell proliferation and inducing apoptosis.

### DNA damage response in CBZ-resistant prostate cancer

**Figure 4:**
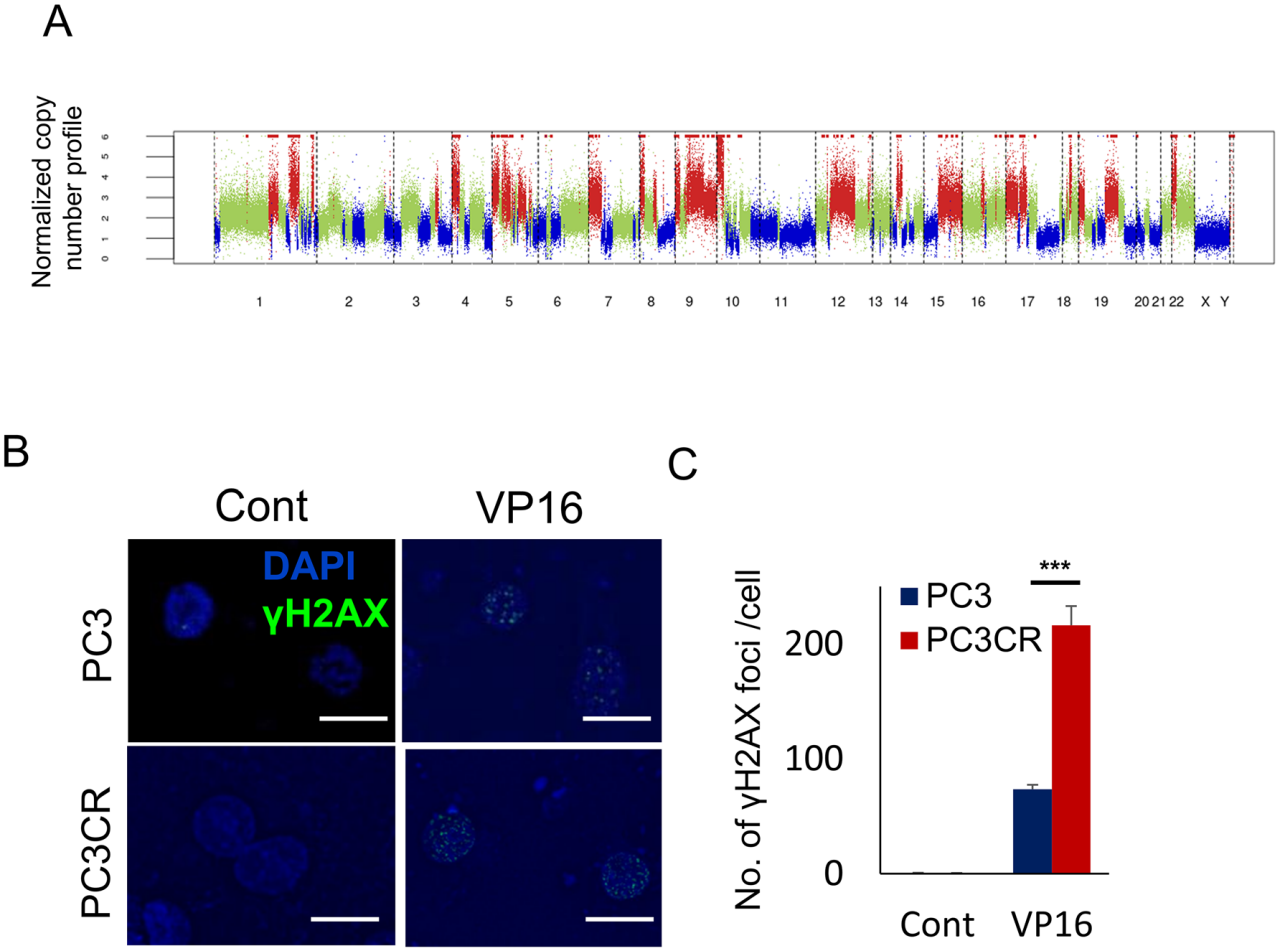
DNA repair pathway in cabazitaxel (CBZ)-resistant prostate cancer. (**A**) Copy number alteration analysis in PC3CR cells. (**B**) Immunohistochemistry of PC3 and PC3CR cells with or without etoposide (VP16) treatment. (**C**) Number of γH2AX foci/cells in PC3 and PC3CR cells with or without VP16 treatment.

To analyze the effectiveness of VP16 against CBZ-resistant cells, we performed exon sequencing of the PC3 and PC3CR genomes. In copy number analysis, large-scale transitions (LST) of PC3CR cells, defined as chromosomal breakages that generate chromosomal gains or losses of greater than or equal to 10 Mb^17^ was 41, and it suggested chromosomal instability in PC3CR cells (Fig. 4A). Genetic alteration was not identified among RB1 nor DNA repair genes in PC3CR. Because VP16 traps TOP2A on DNA strands and induces DNA strand breaks, we evaluated DNA damage induced by VP16 in PC3 and PC3CR cells. Immunofluorescence staining demonstrated that VP16 exposure was linked to higher γH2AX focus counts in PC3CR cell nuclei than in PC3 cell (Fig. 4B,C).

### Targeting DNA repair pathways in CBZ-resistant prostate cancer

**Figure 5:**
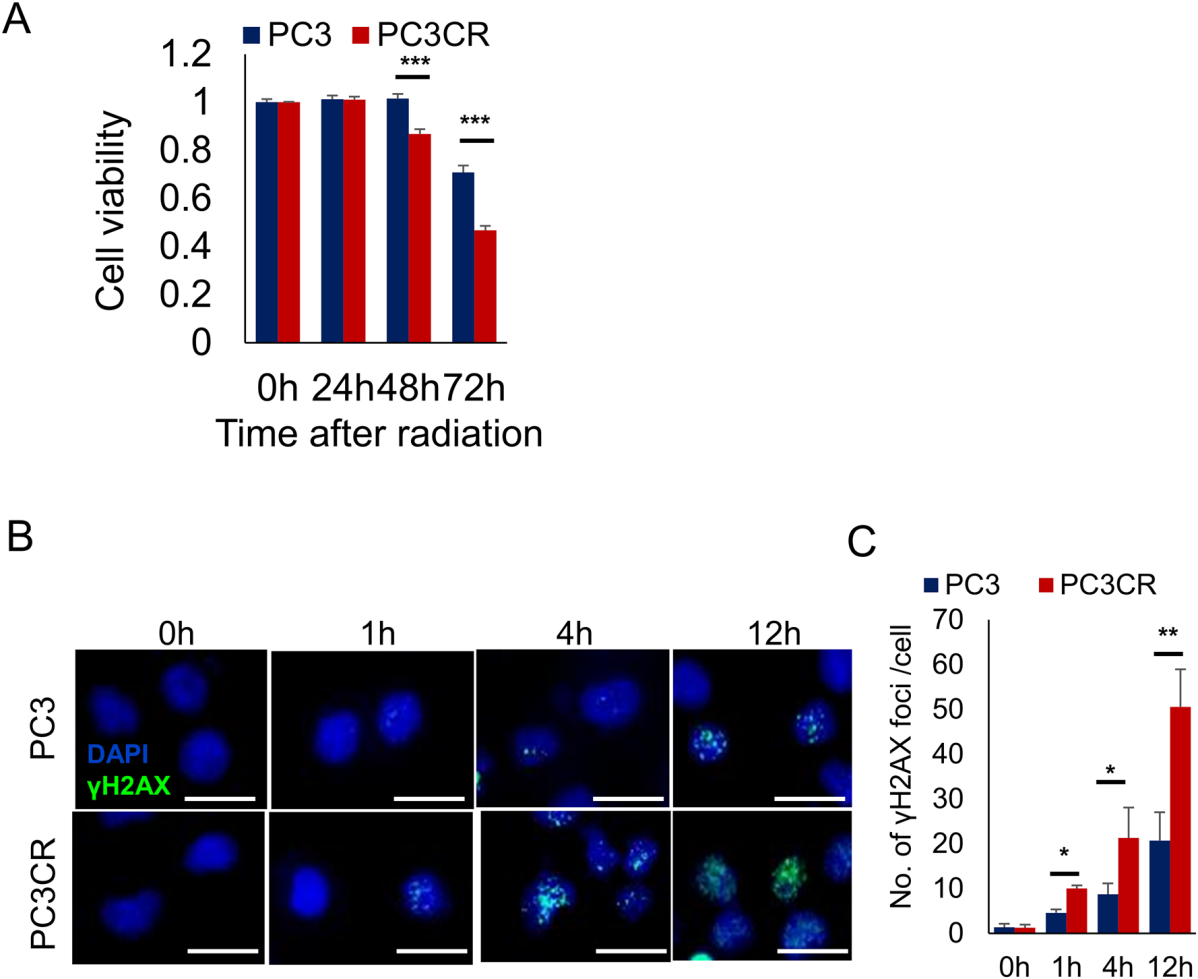
Efficacy of radiation for cabazitaxel (CBZ)-resistant castration-resistant prostate cancer. (**A**) WST assay before and 24, 48, and 72 h after a single 6-Gy dose of radiation in PC3 and PC3CR cells. PC3CR cells exhibited significantly higher radiosensitivity than PC3 cells. (**B**) Immunofluorescence of PC3 and PC3CR cells before and 1, 4, and 12 h after a single 6-Gy dose of radiation. (**C**) Number of γH2AX foci/cells in PC3 and PC3CR cells before and 1, 4, and 14 h after a single 6-Gy dose of radiation.

According to a previous report, the functional loss of genes involved in DNA repair pathways was associated with sensitivity to radiotherapy^18^. To directly investigate the effectiveness of targeting DNA repair pathways in CBZ-resistant cells, we exposed PC3 and PC3CR cells to radiation. Radiation treatment more strongly significantly suppressed proliferation in PC3CR cells than in PC3 cells (Fig. 5A). Immunofluorescence demonstrated that radiation treatment induced greater γH2AX focus formation in PC3CR cell nuclei than in PC3 cell nuclei (Fig. 5B,C). These results suggested that DNA repair pathways could be potential treatment targets for CBZ-resistant prostate cancer.

## Discussion

In this study, we identified a new indication for VP16 for overcoming CBZ-resistant prostate cancer via bioinformatic analysis. VP16 is a derivative of podophyllotoxin from the wild mandrake^19^. Certainly, VP16 also suppressed the proliferation of PC3 cells. However, PC3CR had higher sensitivity to VP16. These results suggested that VP16-based chemotherapy might be an effective option for CBZ-resistant prostate cancer. The agent, which inhibits TOP2A, is used to treat some types of cancers such as lung, blood, and breast cancers^20–22^. VP16 acts as a TOP2 poison through the trapping and stabilization of TOP2-DNA complexes, generating DNA break^23^. In our study, we observed DNA breaks induced by VP16 through γH2AX immunofluorescence. Parental PC3 cells had PTEN/TP53 deletions^24^. PC3CR had no further genetic alteration in tumor suppressor genes nor DDR genes. In addition, neuroendocrine markers were not upregulated in our CBZ-resistant cell line. Beltran et al. reported on prostate cancer lacking both AR and neuroendocrine markers (“double negative” prostate cancer)^25^ and our CBZ-resistant cell lines were classified into this double negative group. However, mechanisms of double negative prostate cancer remained unclear. The lack of AR expression in parental cell lines may be related to the negative neuroendocrine markers. Although PC3 and PC3CR cells are neuroendocrine marker negative and AR signaling negative prostate cancer, VP16 was considered effective for some types of refractory prostate cancer. We also found that targeting DNA repair pathways using radiation was significantly more effective against CBZ-resistant cells than against CBZ-sensitive cells. The accumulation of mutations eventually leads to cancer. Therefore, DNA damage is an important factor for cancer development. Conversely, DNA repair pathwaysos such as BRCA, CHEK, or PALB2 signaling is believed to affect prostate cancer development and progression^26^. Deregulation of SPOP, which is involved in DNA damage responses, was reported to be related to the response to radiotherapy^27^. Remodeling of DNA repair pathways could be potential targets for CBZ-resistant prostate cancer, although further analysis is needed to truly prove involvement of DDR pathways for the acquisition of CBZ resistance.

TOP2A generates double-strand breaks and controls the topological state of DNA strands. The enzyme unlinks supercoils to allow the transcription/replication machinery to access the DNA. The protein expression and catalytic activity of TOP2A depend on the cell cycle stage, specifically peaking in G2/M phase. Because TOP2A has an important role in DNA processing, the deregulation of TOP2A results in inappropriate DNA recombination and chromosomal translocation^28^. Although DNA strand breaks generally lead to cell death, some cells acquire genomic mutations or translocations and progress to malignancy^29,30^. TOP2A upregulation has been linked to tumor progression in breast^31^ and non-small cell lung cancers^32^. Concerning prostate cancer, topoisomerases cooperate with androgen receptor signaling^33–35^. However, the relationship between TOP2A and androgen independence or chemoresistance in prostate cancer is unclear. Our analysis of a prostate cancer cohort suggested that TOP2A expression was associated with tumor progression and chemoresistance. Because VP16 has been used to treat NEPC, we evaluated neuroendocrine markers in prostate cancer cell lines. Whereas NCIH660 tumors expressed CD56, chromogranin A, and synaptophysin, CBZ-resistant tumors did not have significant expression of these markers. Considering these results and previous findings that NEPC has poor responses to taxanes^36^, prostatic adenocarcinoma with acquired CBZ resistance and NEPC might have common characteristics that are correlated with increased sensitivity to VP16. Thus, VP16-based chemotherapy could be a therapeutic option for CBZ-resistant prostate cancer even if NEPC was not detected via tumor biopsy.

Currently, huge expenditures are required for successful novel drug discovery. Our method of drug screening using bioinformatic analysis can permit less costly drug discovery. Because the candidate drugs identified via in silico screening are already used in humans, its side effects and management are well known^5,9,37–39^. We previously identified the hepatitis C drug ribavirin as an option for overcoming DOC resistance in prostate cancer via in silico screening^8^. Based on the same concept as our previous study, we performed in silico drug screening and identified VP16 as a promising candidate treatment for CBZ-resistant prostate cancer. VP16 based chemotherapy may thus be an optimal treatment for CPRC in the post-cabazitaxel setting.

## Supplementary Information

Supplementary Figures.

## Data Availability

The datasets used and/or analysed during the current study are available from the corresponding authors on reasonable request.
