# Supplementary material for: Topoisomerase II alpha inhibition can overcome taxane-resistant prostate cancer through DNA repair pathways
Source: Sci Rep. 2021 Nov 15;11:22284. doi: 10.1038/s41598-021-01697-2 (PMC8593019; doi:10.1038/s41598-021-01697-2)

Supplementary Figure 1. DAVID functional annotation clustering (FAC) analysis of microarray data in PC3CR cells compared with PC3 cells.

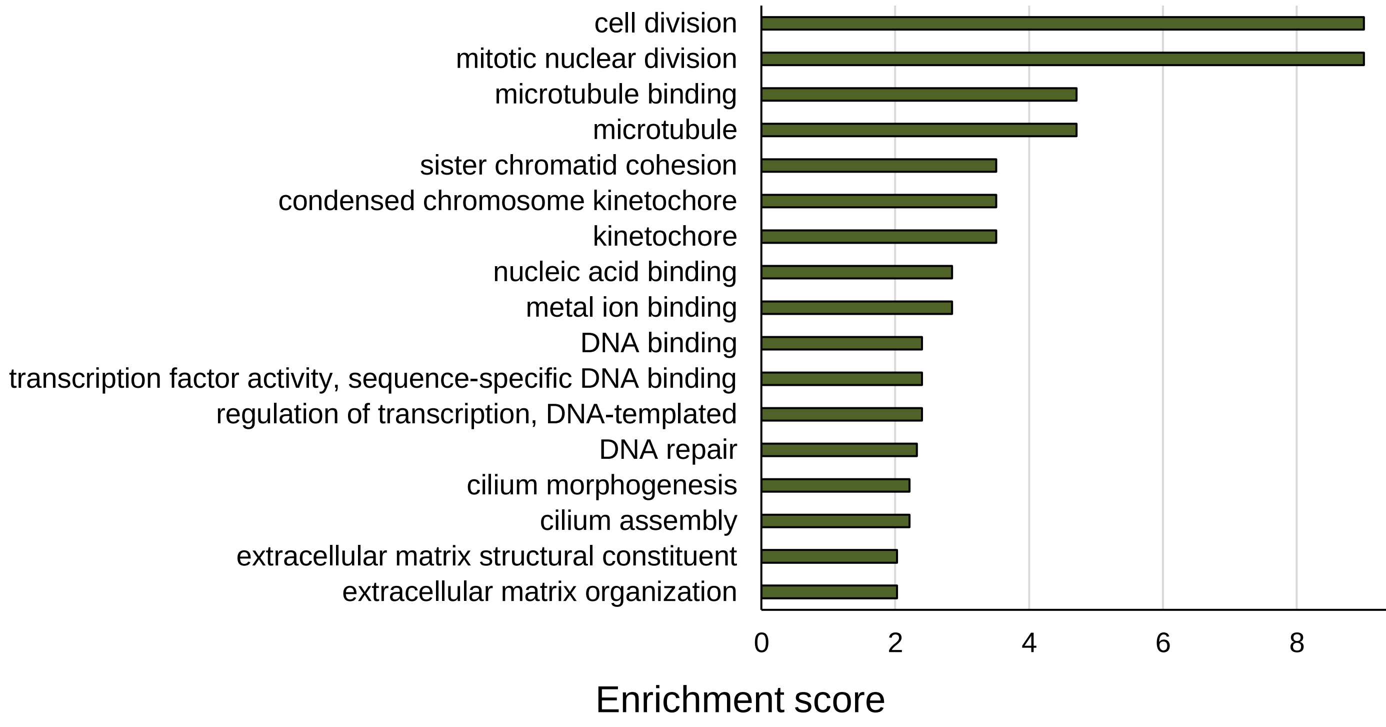

Supplementary Figure 2. *In vitro* screening of candidate drugs to overcome CBZ-resistance in castration-resistant prostate cancer.

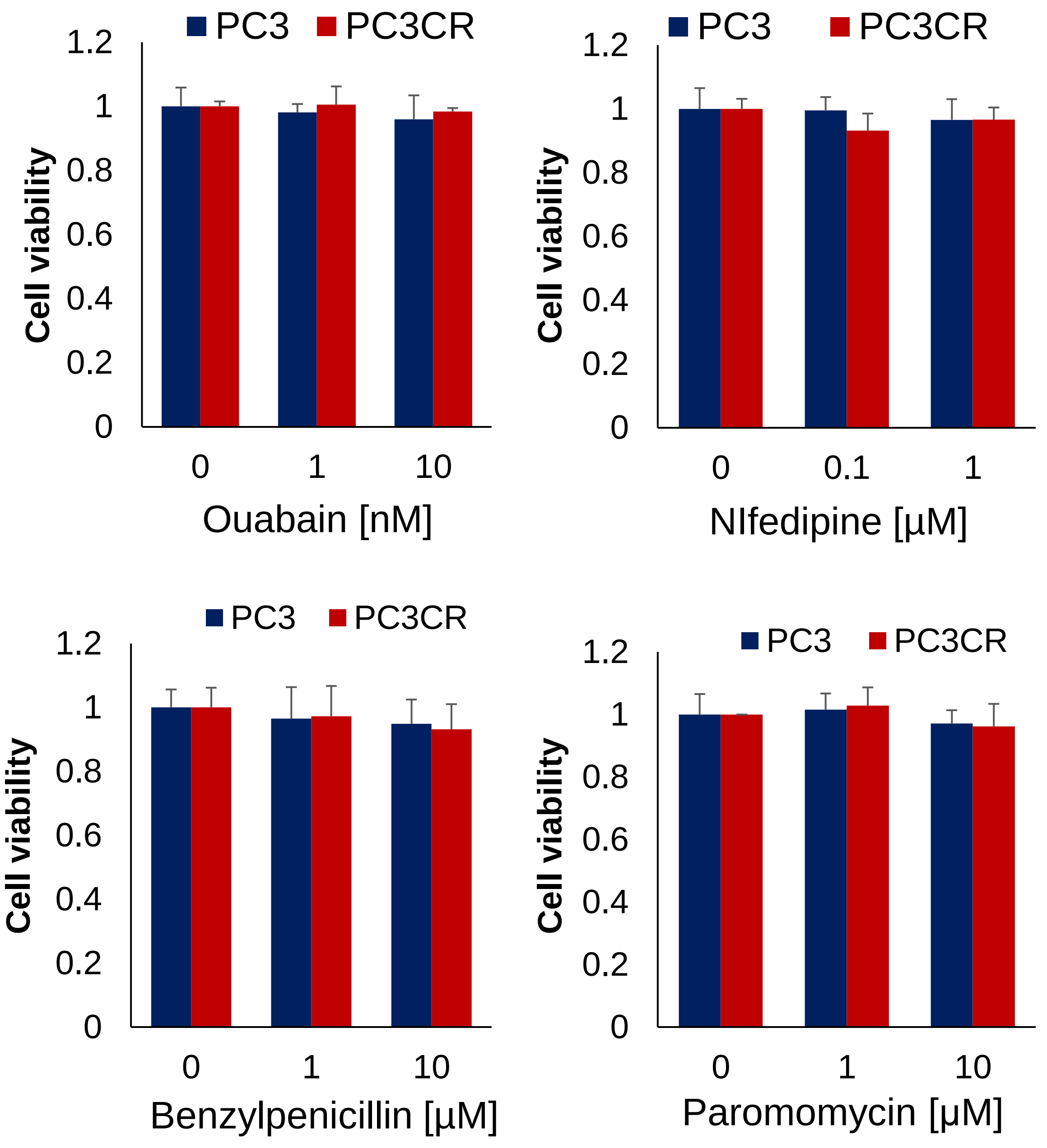

Supplementary Figure 3. Anti-tumor effect of etoposide (VP16) for DU145CR

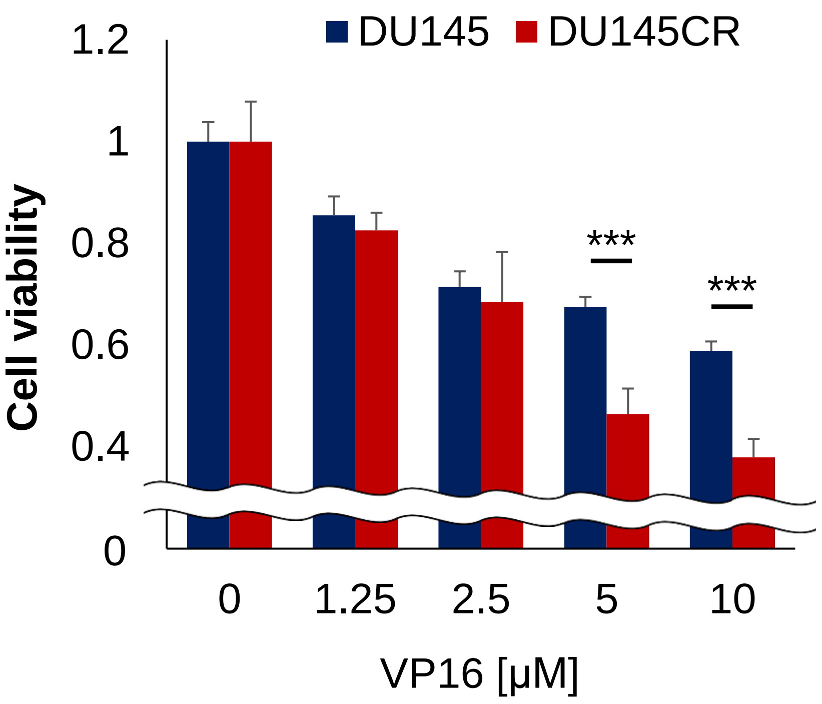

*** p<0.001

Supplementary Figure 4. Neuroendocrine markers expression in xenograft tumors.

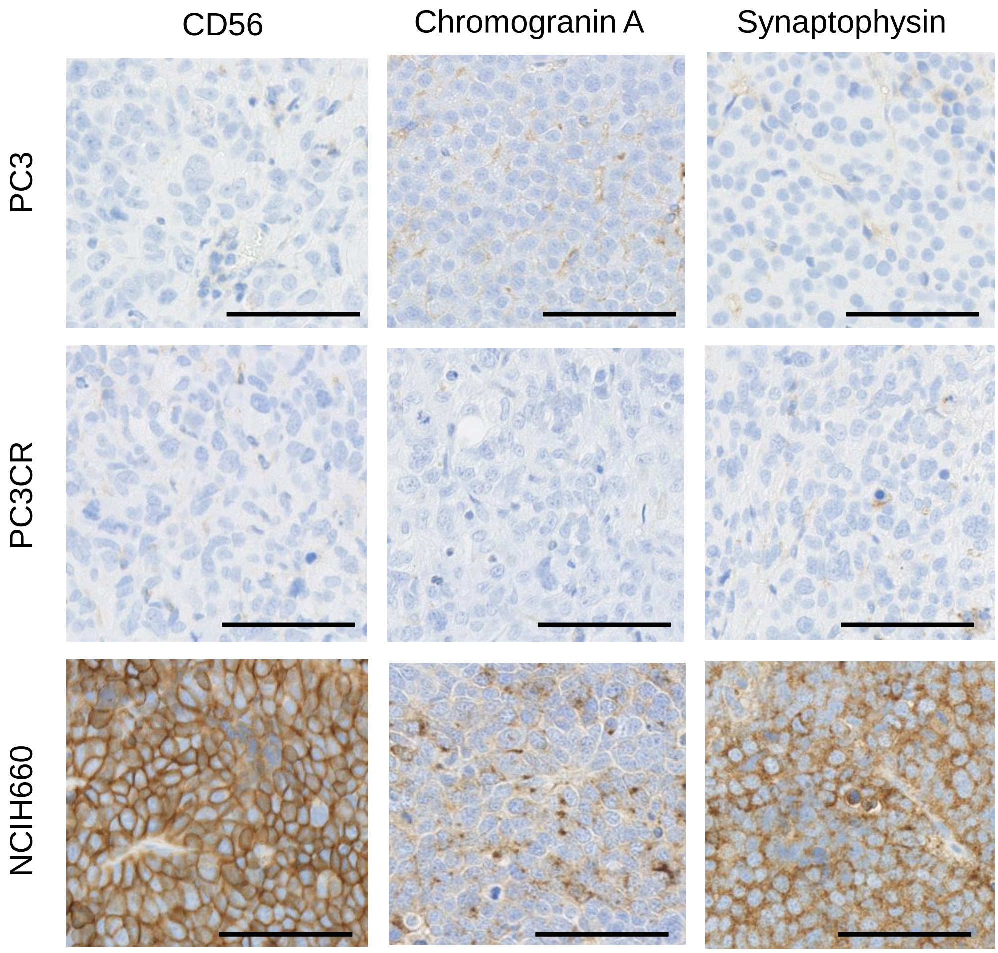

Supplementary Figure 5. Full-length images of western blotting

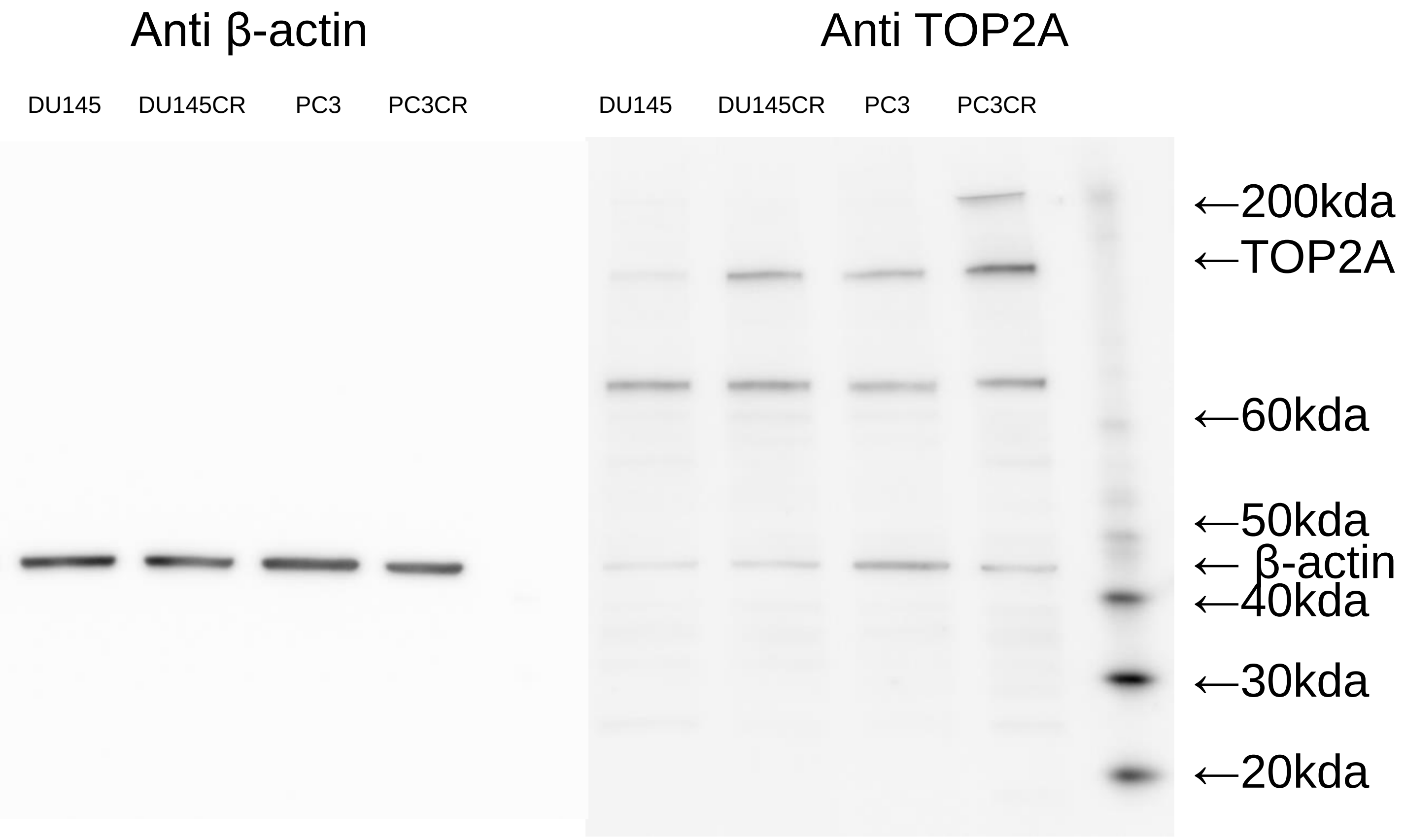

Supplementary Figure 6. TOP2A protein expression in DU145CR

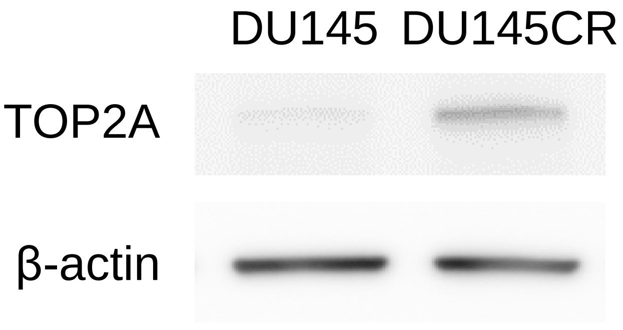

Supplement: Supplementary file 1 — Supplementary Figures. [file 41598_2021_1697_MOESM1_ESM.pdf]
